# The Unifying Nature of Basic Science Research

**DOI:** 10.1371/journal.ppat.1005329

**Published:** 2016-03-31

**Authors:** Kirk W. Deitsch

**Affiliations:** Department of Microbiology and Immunology, Weill Medical College of Cornell University, New York, New York, United States of America; University of Notre Dame, UNITED STATES

Why does research matter? Why do professional scientists choose to spend their careers working on specific topics, and what impact does this have on society and the people (largely taxpayers) who fund the research enterprise? Is the public investment in many diverse areas of fundamental research really worth it? These are questions that I have asked myself throughout my career as I have progressed from student to research fellow to head of an academic laboratory. My experience has convinced me of the value of broad-based, highly diverse basic research.

Like most molecular biologists, in my early training I was exposed to a wide variety of experimental systems, from mammalian cell culture to fruit flies to mice. While I found all of these models interesting and well worth studying, it was when I was first exposed to the complex interplay between pathogens and their hosts that I became utterly captivated. In particular, I was fascinated by the intricate methods pathogens use to systematically vary the proteins they expose to their host’s immune system, a process referred to as antigenic variation. Virtually all pathogens that are able to maintain chronic infections have evolved the ability to display only a limited number of molecules that the host is able to recognize and target for the development of an immune response. Then, just when the immune system begins to gain the upper hand and eliminate the infection, the pathogen changes its surface coat and displays an entirely new set of antigens that the host must respond to; thus, the whole process starts again. Many of these pathogens maintain large repertoires of genes that encode these “disguises,” utilizing a complex process of coordinated gene activation and silencing that enables them to slowly reveal each variant one at a time over the course of an infection. As a research subject, this topic has many interesting angles: coevolution of two organisms, complex networks of gene expression, antigen recognition and diversification, to name a few. Once this game of molecular “hide and seek” between host and pathogen was revealed to me, I was hooked, and I have continued to work on this topic throughout my career.

Our work to understand the mechanisms underlying antigenic variation in eukaryotic pathogens has led us to many of the same molecular processes that have recently been discovered in the study of eukaryotic model systems. For example, many of the gene regulatory mechanisms, in particular epigenetic processes, that higher eukaryotes utilize to control gene expression during cellular differentiation are used by parasites and pathogenic fungi to control the expression of the genes encoding variant surface antigens. In higher eukaryotes, these epigenetic modifications are often part of the terminal differentiation process and, thus, are typically permanent, while in pathogens the changes are reversible, thereby enabling them to switch genes on and off and adding a clever twist to the story. I am fascinated by the parallels in the molecular mechanisms employed by both host and pathogen to regulate gene expression. Working in this field has brought me back to the early days of my training as a molecular biologist when my studies focused on model eukaryotic systems, and today I am just as likely to read a paper about zebra fish as I am about malaria parasites, the focus of my research.

The shared mechanisms of epigenetic gene regulation employed both by higher eukaryotic organisms (including humans) and infectious pathogens reminds us of the unifying nature of fundamental, basic research in the biological sciences. Concepts deciphered through studies of yeast, fruit flies, slime molds, or the simple worm *Caenorhabditis elegans* can have profound implications for our understanding of human biology or the pathogens that infect us. This synergism between fields of study greatly increases the pace of discovery in all systems and provides a reminder that, as scientists, we are all on the same team. As a researcher works diligently to understand a key aspect underlying how a cell in a fruit fly becomes part of a leg versus an antenna, he or she might be making a key discovery that will influence the development of a novel drug to treat malaria, a disease that kills hundreds of thousands of children annually. The march of science is unpredictable, and it is often difficult to imagine where the next breakthrough will come from. Nonetheless, we can be sure that diverse, fundamental, basic science will continue to improve the lives of people all over the planet, just as it has for centuries. In my mind, this is why research matters.

**Image 1:**
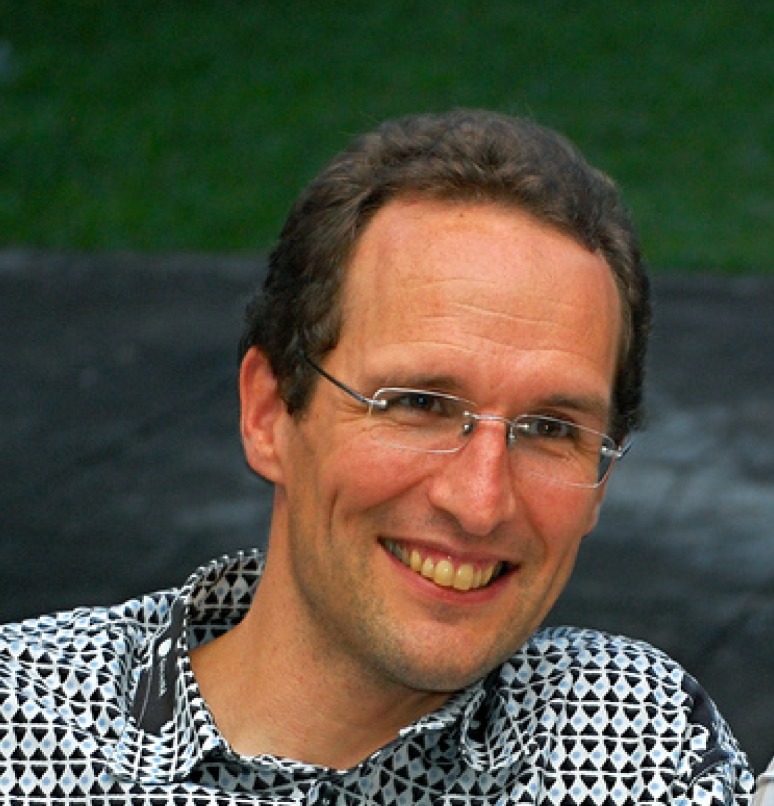
Kirk W. Deitsch.

